# 3-month surgical outcomes of Implantable Collamer Lens implantation for myopic regression after laser vision correction surgeries: a retrospective case series

**DOI:** 10.1186/s12886-021-02163-3

**Published:** 2021-11-16

**Authors:** Byunghoon Chung, Joon Hyun Kim, David S. Y. Kang, Dong Jun Kang, Eung Kweon Kim, Kyoung Yul Seo, Ikhyun Jun, Tae-im Kim

**Affiliations:** 1 Eyereum Eye Clinic, Seoul, Republic of Korea; 2 Apgujeong Eye Clinic, Seoul, Republic of Korea; 3 Department of Ophthalmology, Institute of Vision Research, Yonsei University College of Medicine, 50-1, Yonsei-ro, Seodaemun-gu, Seoul, 03722 Republic of Korea

**Keywords:** Implantable collamer lens, Myopic regression, Laser-assisted in situ keratomileusis, Photorefractive keratectomy

## Abstract

**Background:**

To investigate the surgical outcomes of implantable collamer lens (ICL) implantation in eyes with residual myopia after primary laser vision correction (LVC) surgeries.

**Methods:**

This study included patients who underwent ICL implantation and had a history of LVC surgery, including photorefractive keratectomy (PRK) or laser-assisted in situ keratomileusis (LASIK). Visual acuity and refractive error were assessed pre and 3-months postoperatively and the efficacy and safety indices calculated accordingly.

**Results:**

A total of 30 eyes of 17 patients were included in this study. At 3 months, the mean logMAR uncorrected distance visual acuity (UDVA), corrected distance visual acuity (CDVA), and spherical equivalent were − 0.03 ± 0.11 (include logMAR), − 0.04 ± 0.09 (include logMAR), and − 0.06 ± 0.33 diopters (D), respectively. The 3-month Snellen UDVA was better than 20/20 for 83% of eyes, and 97% of eyes showed an unchanged or improved CDVA after surgery. The mean efficacy and safety indices were 1.11 ± 0.22 and 1.13 ± 0.20, respectively. Further, 93 and 100% of eyes were within ±0.5 and ± 1.0 D of the attempted spherical equivalent refraction, respectively.

**Conclusions:**

ICL implantation in eyes with myopic regression after previous LVC surgery showed safe, effective, and predictable outcomes.

**Trial registration:**

retrospectively registered.

## Background

Regression following corneal refractive procedures refers to the tendency of the cornea to shift towards the preoperative refractive status after achieving desired refraction for a period of time [1]. Residual refractive errors after primary laser vision correction (LVC) surgery is the most common cause of postoperative dissatisfaction, and surgical retreatment is considered in eyes with regression. The rate of enhancement, the surgical retreatment, has been reported to be 1.8–22% for laser-assisted in situ keratomileusis (LASIK) [2–4] and 2.3–5.2% for photorefractive keratectomy (PRK) [5, 6].

Enhancement options for LASIK include flap re-lift with ablation or recutting a new flap and surface ablation, and for PRK include LASIK or a new PRK procedure. However, these enhancement options are only available when the residual stromal bed is thick enough to maintain corneal biomechanical stability after the enhancement procedure. In addition, enhancement options involving corneal laser procedures pose a risk of postoperative complications, including epithelial ingrowth, subepithelial haze formation, and corneal ectasia.

Posterior chamber phakic intraocular lens implantation has been reported to be safe and effective for the correction of myopia and myopic astigmatism throughout a long-term follow-up period [7]. The implantable collamer lens (ICL) is widely used to correct moderate and high myopia, and some studies have reported ICL implantation in eyes that underwent previous corneal surgeries, including LASIK, PRK, and radial keratotomy [8–10]. Among these studies, one reported ICL implantation to be a safe and effective way to retreat residual myopia after primary corneal refractive surgeries [8]. In the present study, we aimed to analyze the surgical outcomes of ICL implantation in eyes with myopic regression after primary LVC surgeries including LASIK and PRK.

## Methods

### Study design

This retrospective, observational case series was conducted at Yonsei University College of Medicine, Seoul, Republic of Korea. The tenets of the Declaration of Helsinki and good clinical practice were followed, and institutional review board approval from Yonsei University College of Medicine was obtained (No. 4–2020-0396). Owing to the retrospective nature of the study, the requirement for informed consent was waived. All surgeries were performed by a single surgeon (JHK) between June 2020 and August 2020.

### Study population

A total of 17 patients with residual myopia or myopic astigmatism after a previous history of LVC were enrolled. All patients decided to have ICL implantation after detailed explanation of possible enhancement options before the surgery. Other inclusion criteria were a stable refractive status for at least 1 year, a preoperative corrected distance visual acuity (CDVA) of 20/30 or better, and no sign of corneal ectasia on at least three consecutive corneal tomographic evaluations. Patients with any kind of ocular surface diseases, ocular trauma, glaucoma, cataract, an endothelial cell density < 2000 cells/mm^2^, or an anterior chamber depth from the endothelium < 2.8 mm were excluded.

### Assessment

To evaluate outcomes, all patients were assessed before and 3 months after ICL implantation. Patients’ assessments included uncorrected distance visual acuity (UCVA) and CDVA measured in logMAR, slit-lamp examination (Haag-Streit AG), keratometry, pachymetry (ARK-530A Auto Ref/Keratometer, Nidek Co., Ltd.), specular microscopy (SP-3000P, Topcon Corporation), Scheimpflug-based corneal tomography (Pentacam HR, OCULUS Optikgeräte GmbH), and anterior segment optical coherence tomography (Visante, Carl Zeiss Meditec AG). The efficacy index (the ratio of postoperative UDVA to preoperative CDVA) and safety index (the ratio of postoperative CDVA to preoperative CDVA) were also estimated.

### Surgical procedure

All procedures were performed through a superior 3.0-mm corneal incision after instillation of 0.5% phenylephrine and 0.5% tropicamide (Mydrin-P, Santen Pharmaceutical Co., Ltd.) under topical anesthesia with 0.5% proparacaine (Alcaine, Alcon). Further, 1% sodium hyaluronate (Healon, Johnson & Johnson Vision) was injected into the anterior chamber, and the ICL was inserted through an injector cartridge. After positioning the ICL, the sodium hyaluronate was completely removed by manual irrigation and aspiration. The V4c ICL model (Staar Surgical) was used in all cases. ICL powers were calculated using a modified vertex formula provided by the manufacturer. Patients were instructed to use 0.5% moxifloxacin (Vigamox, Alcon) and 1% prednisolone (Pred Forte, Allergan, Inc.) four times a day for a week.

### Statistical analysis

Data are presented as mean ± standard deviation. IBM SPSS Statistics for Windows (v. 25.0, IBM Corporation) was used to perform statistical analyses. Data normality was confirmed using a Kolmogorov–Smirnov test. Pre- and postoperative measurements were compared using a paired t-test for normally distributed data and the Wilcoxon signed-rank test for non-normally distributed data. Comparisons between the two groups were performed using an independent sample t-test for normally distributed data and a Mann–Whitney U test for non-normally distributed data. The refractive predictability was analyzed using linear regression, and comparison of categorical variables was performed using a chi-squared test. A *P*-value < .05 was considered statistically significant.

## Results

### Patient characteristics

A total of 30 eyes of 17 patients were enrolled in the present study. Patient characteristics are presented in Table 1. The mean central corneal thickness, mean keratometry reading, and endothelial cell count were not significantly changed at 3 months after ICL implantation compared to those before the implantation. There were no intraoperative and postoperative complications, including cataract formation and elevation of intraocular pressure, in any of the cases.

**Table 1:** Characteristics of eyes which underwent implantable collamer lens implantation after previous laser vision correction surgery

Characteristics	Preop	3-month postop	***P***
Age (years)	35.00 ± 4.48 (27 to 43)		
No. of eyes (right/left)	15/15		
Sex (M/F)	8/22		
Type of previous surgery (PRK/LASIK)	21/9		
Time interval after previous surgery (months)	73.27 ± 6.70 (61 to 84)		
Central corneal thickness (μm)	456.03 ± 35.71 (413 to 524)	456.77 ± 35.48 (412 to 520)	0.647
Mean keratometry (D)	38.96 ± 1.39 (35.63 to 41.13)	38.90 ± 1.52 (35.13 to 41.88)	0.295
Endothelial cell count (/mm^2^)	3006 ± 290 (2480 to 3524)	2999 ± 367 (2314 to 3771)	0.841
LogMAR UDVA	0.70 ± 0.33 (0.10 to 1.30)	−0.03 ± 0.11 (− 0.18 to 0.30)	< 0.001*
LogMAR CDVA	0.01 ± 0.04 (0 to 0.15)	− 0.04 ± 0.09 (− 0.18 to 0.22)	0.001*
Refractive errors (D)			
Sphere	−2.32 ± 1.21 (−6.00 to − 0.75)	0.05 ± 0.31 (− 0.50 to 0.75)	< 0.001*
Cylinder	−0.37 ± 0.39 (−1.00 to 0)	−0.23 ± 0.40 (− 1.25 to 0)	0.084
Spherical equivalent	−2.50 ± 1.22 (− 6.25 to − 1.13)	− 0.06 ± 0.33 (− 0.63 to 0.75)	< 0.001*

*PRK* photorefractive keratectomy; *LASIK* laser in situ keratomileusis; *D* diopters; *UDVA* uncorrected distance visual acuity; *CDVA* corrected distance visual acuity; Values are presented as mean ± standard deviation (range) * significant difference between preop and postop 3-month measurements

### Visual outcomes, efficacy, and safety

**Fig. 1:**
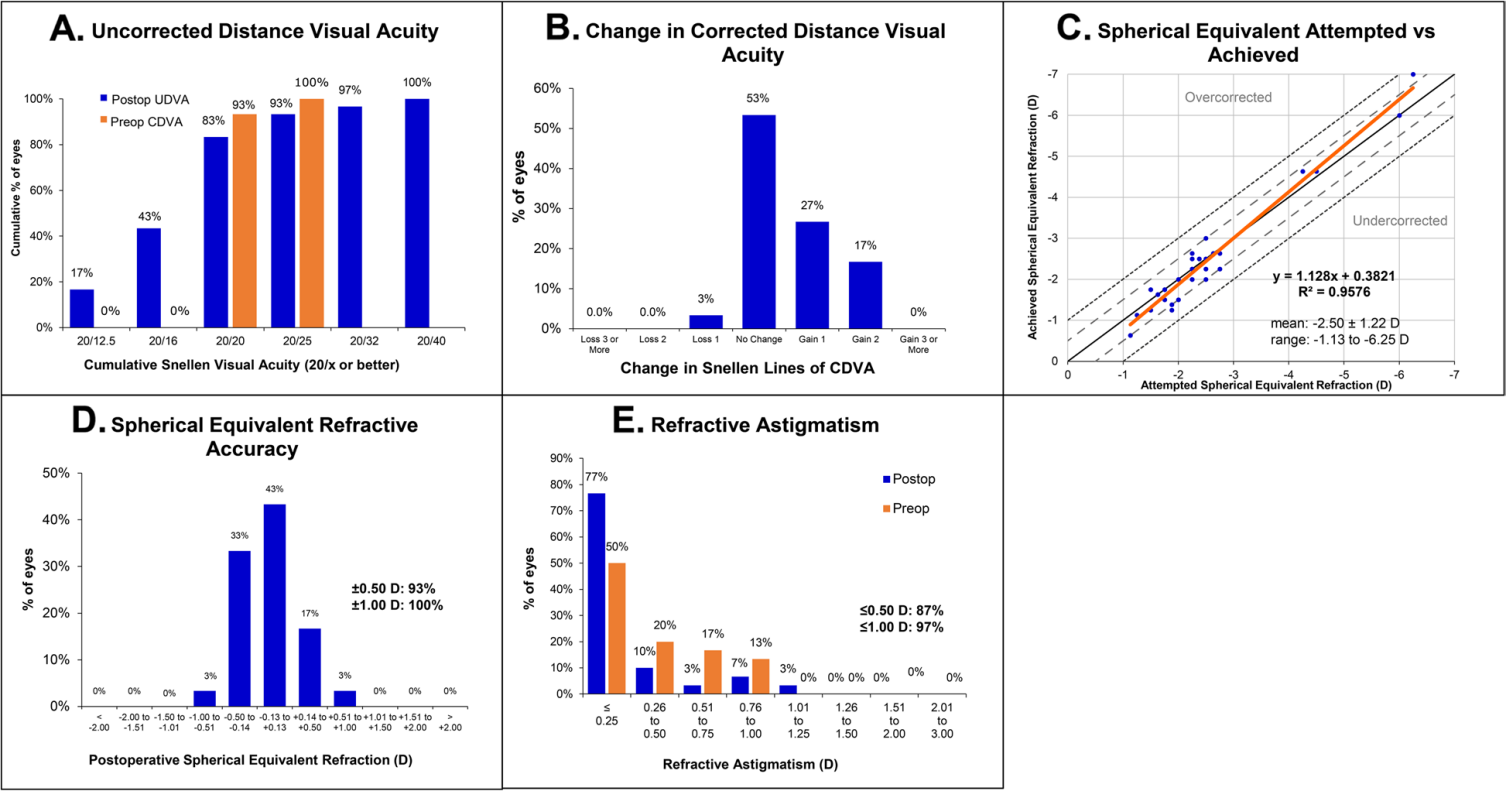
Visual and refractive outcomes after implantable collamer lens implantation (ICL) in eyes with myopic regression after primary laser vision correction surgery. **A** Cumulative 3-month postoperative uncorrected distance visual acuity (UDVA) and preoperative corrected distance visual acuity (CDVA). **B** Changes in Snellen lines of postoperative CDVA relative to the preoperative CDVA. **C** The attempted versus achieved changes in spherical equivalent refraction (SEQ) 3 months after ICL implantation. **D** Accuracy of SEQ relative to the intended target. **E** Preoperative and postoperative refractive astigmatism. D = diopters

The mean UDVA and CDVA improved significantly at 3 months after ICL implantation (Table 1); 83% of eyes showed a 3-month UDVA of 20/20 or better (Fig. 1A). One eye showed loss of one Snellen line of CDVA at 3 months after ICL implantation, while the remaining eyes showed no change or gain of Snellen lines of CDVA (Fig. 1B). The mean efficacy index (ratio of postoperative UDVA to preoperative CDVA) and safety index (ratio of postoperative CDVA to preoperative CDVA) were 1.11 ± 0.22 and 1.13 ± 0.20, respectively. Additional analysis was performed according to the types of primary LVCs (PRK and LASIK; Table 2). There was no significant difference between the PRK and LASIK groups regarding UDVA, CDVA, and refractive errors.

**Table 2:** Characteristics of eyes which underwent implantable collamer lens implantation after previous laser vision correction surgery according to the types of previous laser vision correction

Type of previous LVC	PRK (***n*** = 21)	LASIK (***n*** = 9)	***P***
No. of eyes (right/left)	11/10	4/5	> 0.999
Age (years)	33.38 ± 4.04	38.78 ± 3.00	0.001*
Sex (M/F)	6/15	2/7	> 0.999
Time interval after previous surgery (months)	71.29 ± 5.82	77.89 ± 6.59	0.022*
Central corneal thickness (μm)	452.95 ± 34.69	463.22 ± 39.12	0.455
Preoperative mean keratometry (D)	38.97 ± 1.50	38.94 ± 1.18	0.700
LogMAR UDVA			
Preoperative	0.70 ± 0.28	0.69 ± 0.43	0.982
3-month postoperative	−0.04 ± 0.07	0 ± 0.16	0.657
*P* (preop vs 3-month postop)	< 0.001	0.008	
LogMAR CDVA			
Preoperative	0	0.03 ± 0.07	0.349
3-month postoperative	−0.05 ± 0.06	0 ± 0.14	0.563
*P* (preop vs 3-month postop)	0.006	0.114	
Spherical error (D)			
Preoperative	−2.00 ± 0.48	−3.08 ± 1.95	0.504
3-month postoperative	−0.02 ± 0.28	0.22 ± 0.32	0.051
*P* (preop vs 3-month postop)	< 0.001	0.008	
Cylindrical error (D)			
Preoperative	−0.37 ± 0.42	− 0.36 ± 0.33	0.981
3-month postoperative	− 0.24 ± 0.39	−0.19 ± 0.27	0.918
*P* (preop vs 3-month postop)	0.146	0.301	
Spherical equivalent (D)			
Preoperative	−2.17 ± 0.42	−3.26 ± 2.00	0.733
3-month postoperative	−0.14 ± 0.31	0.13 ± 0.30	0.050
*P* (preop vs 3-month postop)	< 0.001	0.008	
Endothelial cell count (/mm^2^)			
Preoperative	2986 ± 314	3053 ± 233	0.556
3-month postoperative	2966 ± 388	3076 ± 321	0.319
*P* (preop vs 3-month postop)	0.476	0.859	

*PRK* photorefractive keratectomy; *LASIK* laser in situ keratomileusis; *D* diopters; *UDVA* uncorrected distance visual acuity; *CDVA* corrected distance visual acuity; Values are presented as mean ± standard deviation (range) * significant difference between previous PRK and LASIK group

### Refraction and predictability

Refractive errors, including sphere and spherical equivalent (SEQ), decreased significantly at the 3-month postoperative evaluation, while cylindrical errors showed no significant change (Table 1). The attempted versus achieved SEQ graph showed a slope and correlation coefficient (R^2^) of 1.13 and 0.96, respectively (Fig. 1C). In terms of refractive accuracy, 93 and 100% of eyes showed an achieved SEQ within ±0.50 diopters (D) and ± 1.0 D, respectively (Fig. 1D). The preoperative refractive astigmatism was within 0.50 D and 1.00 D in 70 and 100% of eyes, respectively, while the postoperative astigmatism was within 0.50 D and 1.00 D in 87 and 97% of eyes, respectively (Fig. 1E).

## Discussion

This study presented ICL implantation to be an effective, safe, and predictable method to treat myopic regression in eyes underwent previous LVC. 3-month postoperative mean UDVA and CDVA were significantly improved after ICL implantation, and 97% of eyes showed no change or gain of Snellen lines of CDVA. Also, 93% of eyes showed an achieved SEQ within ±0.50 D. Several mechanisms are suggested to be related to myopic regression after LVC, but the exact mechanism leading to regression is yet to be elucidated. Among these mechanisms, corneal epithelial remodeling and corneal stromal healing response are major factors for myopic regression [1, 11, 12]. Compensatory epithelial hyperplasia and a forward shift of both anterior and posterior corneal surfaces can lead to myopic regression following LVC.

Secondary corneal surgeries to treat myopic regression includes the flap re-lift LASIK and surface ablation [13]. These types of surgeries can induce aggressive corneal wound healing response and pose a risk of epithelial ingrowth and corneal haze formation. Furthermore, increased risk of corneal ectasia and induction of higher-order aberrations can negatively affect the outcomes of the procedure. Surgical retreatment using phakic intraocular lens implantation can naturally avoid corneal complications. Fast visual recovery and relatively less postoperative pain are also expected. Several studies have reported the outcomes of phakic intraocular lens implantation after previous corneal surgeries, including radial keratotomy, LASIK, and PRK [8, 10, 14, 15]. ICL implantation was reportedly effective for both myopic and hyperopic regression. Stable refractive outcomes up to 5 years after ICL implantation was also reported in one of the studies [8]. In our study, ICL implantation in eyes with myopic regression showed safe, effective, and predictable outcomes in larger sample compared to the previous study [8]. The 3-month Snellen UDVA was better than 20/20 for 83% of eyes, and the safety and efficacy indices at 3 months postoperatively were 1.13 ± 0.20 and 1.11 ± 0.22, respectively. These results were similar to or better than those of a previous study in which 52.6% of eyes showed a UDVA better than 20/20 at 1 week–1 month postoperatively [8]. In addition, there was no clinically significant difference in the postoperative outcomes according to the type of primary LVC surgery including PRK and LASIK. As the size of each group was relatively small, generalizing these results is difficult. Further investigation in a large sample will be helpful to investigate results of enhancement by ICL implantation according to types of primary LVC surgery. Calculation of ICL power is based on the vertex formula modified by Feingold and Olsen [16–18]. Mean corneal power, corneal thickness and the anterior chamber depth are needed to calculate the power of the ICL. As the ratio of the anterior and posterior corneal curvature changes after LVC surgery, it could be a source of error in ICL power calculation. Despite previous corneal surface modification, the modified vertex formula showed predictable outcomes; 93 and 100% of eyes showed an SEQ within ±0.5 and ± 1.0 D, respectively. There was no significant change in the cylindrical error after ICL implantation. This may be owing to the fact that toric ICL was not implanted in any of the cases in our study. A probable source of the refractive astigmatism modification is the surgical induced astigmatism.

Limitations of the present study include a short follow-up period and small sample size. We only included patients with stable refractive errors for at least 1 year to clinically exclude myopic progression, however, there is possibility that myopic progression cases were included as our study did not include evaluation of keratometry and axial length changes before and after the primary LVC surgery. Other assessment modalities, including assessment of wavefront aberrations, will be helpful to analyze the outcomes of ICL implantation after LVC surgeries.

## Conclusions

ICL implantation showed safe, effective, and predictable postoperative outcomes in eyes with myopic regression. In addition, there was no significantly different result based on the type of previous LVC surgery, including PRK and LASIK. ICL implantation can be considered as an appropriate option to treat myopic regression after LVC surgery.

## Data Availability

Not applicable.
